# Functional Characterization of Purinergic Signaling in HOG Human Oligodendroglioma Cell Line: Tumor-Suppressive Role of the P2Y_1_ Receptor

**DOI:** 10.3390/biology15211969

**Published:** 2026-11-02

**Authors:** Inés Baeza-Zaragoza, Celia Llorente-Sáez, Raquel Pérez-Sen, Marina Arribas-Blázquez, Luis Alcides Olivos-Oré, Antonio R. Artalejo, Rosa Gómez-Villafuertes, Felipe Ortega

**Affiliations:** 1 Departamento de Bioquímica y Biología Molecular, Universidad Complutense de Madrid (UCM), 28040 Madrid, Spaincelillor@ucm.es (C.L.-S.); rpsen@ucm.es (R.P.-S.); 2 Instituto Universitario de Investigación en Neuroquímica (IUIN), 28040 Madrid, Spain; marrib01@ucm.es (M.A.-B.); olivos@ucm.es (L.A.O.-O.); artalejo@ucm.es (A.R.A.); 3 Instituto de Investigación Sanitaria San Carlos (IdISSC), 28040 Madrid, Spain; 4 Departamento de Farmacología y Toxicología, Facultad de Veterinaria, Universidad Complutense de Madrid, 28040 Madrid, Spain

**Keywords:** purinergic signaling, P2Y_1_ receptor, oligodendroglioma, HOG cells, proliferation, migration, cell death

## Abstract

Oligodendrogliomas are diffuse low-grade gliomas of the adult central nervous system characterized by high recurrence rates and limited therapeutic options. Extracellular nucleotide signaling via purinergic receptors (P2X and P2Y) regulates pathophysiological processes in various brain tumors; however, its specific role in oligodendrogliomas remains unexplored. Here, we characterized the purinergic receptor profile in HOG cells, a human oligodendroglioma cell line, and evaluated the specific impact of P2Y_1_ receptor activation on tumor cell dynamics. Single-cell calcium imaging experiments demonstrated that extracellular ATP increases intracellular calcium concentration through both extracellular influx and internal store mobilization. Expression analyses at transcript and protein levels identified ionotropic P2X4 and P2X5 receptors, alongside metabotropic P2Y_1_, P2Y_2_, and P2Y_4_ receptors. Furthermore, the calcium responses induced by selective P2Y agonists confirmed functional responses for P2Y_1_ and P2Y_2_/P2Y_4_ receptors. Finally, long-term time-lapse videomicroscopy combined with single-cell lineage tracking revealed that P2Y_1_ activation reduced cell proliferation, prolonged cell cycle duration, tended to reduce migratory velocity, and triggered cell death. Pre-incubation with the selective P2Y_1_ antagonist MRS2179 completely abrogated these effects. Taken together, our findings provide the first evidence of a functional purinergic network in HOG human oligodendroglioma cell line and highlight the P2Y_1_ receptor as a tumor-suppressive candidate and potential therapeutic target for oligodendroglioma management.

## Simple Summary

Oligodendrogliomas are aggressive brain tumors that mainly strike young adults and frequently return after surgery because they grow near critical brain regions responsible for vital functions. Current treatment options remain limited, highlighting an urgent need for novel therapeutic targets. In this study, we investigated whether tumor cells communicate through chemical energy signals in their environment and whether disrupting this communication could stop tumor growth. By examining human brain tumor cells in the laboratory, we discovered that these cells express specific docking proteins on their surface that sense chemical signals. Activating one specific docking protein, known as the purinergic P2Y-one receptor, markedly reduced the ability of tumor cells to multiply, slowed down their movement, and directly triggered tumor cell death. Blocking this same protein allowed the cells to divide even faster, showing that this signaling pathway naturally acts as a brake on tumor progression. These findings demonstrate for the first time that activating this molecular pathway can halt tumor growth, offering a promising strategy to develop targeted drugs that could improve survival rates and quality of life for patients facing brain cancer.

## 1. Introduction

Gliomas are brain tumors that arise from glial progenitor cells [1] and constitute the most frequent primary malignant solid tumors of the adult central nervous system (CNS) [2,3,4]. The term “glioma” encompasses various tumor subtypes, including astrocytomas, oligodendrogliomas, medulloblastomas, Schwannomas, ependymonas, and glioblastomas. These tumors are characterized by their high incidence, poor prognosis, and frequent recurrence [5]. According to the 5th edition of the World Health Organization Classification of CNS Tumors (WHO 2021), adult-type diffuse gliomas are classified into four grades based on specific histological and molecular features [6]. Within this classification, oligodendrogliomas (WHO grades 2 and 3) account for approximately 5% of all primary CNS tumors and predominantly affect young adults in their third to fourth decades of life [7,8]. Although these tumors typically exhibit a more indolent early clinical course compared to glioblastomas, low-grade oligodendrogliomas inevitably undergo malignant transformation into higher, more aggressive histological grades [8,9]. The current standard treatment for oligodendroglioma typically involves maximal safe surgical resection followed by postoperative radiotherapy [10]. However, the preferential localization of oligodendrogliomas in the frontal lobe substantially limits the extent of resection due to the heightened risk of inflicting permanent neurological deficits by damaging critical functional areas of the brain regions [11]. This limitation increases the likelihood of postoperative deficits and negatively impacts patients’ neurological function and quality of life. In cases of tumor recurrence, reoperation and chemotherapy remain the main therapeutic strategies, although their clinical effectiveness is often modest and associated with poor long-term outcomes [11]. These challenges contribute to reduced treatment efficacy and overall survival, underscoring the urgent necessity to identify novel molecular targets and signaling pathways underlying the biology of these tumors.

In recent decades, purinergic signaling mediated by extracellular nucleotides has emerged as a key regulator in CNS physiology and various pathological states, including trauma, ischaemia, epilepsy, neuropsychiatric and mood disorders, neurodegenerative diseases, and cancer, including gliomas [12,13,14,15]. In the tumor microenvironment, extracellular nucleotides are released via both vesicular exocytosis [16,17] and non-vesicular pathways, such as connexin and pannexin hemichannels, ABC transporters, and voltage-dependent anion channels [18,19]. Following their release, nucleotides are rapidly degraded by a cascade of membrane-bound ectonucleotidases [20], generating a dynamic pool of physiological ligands for various purinergic receptors. The presence of ectonucleotidases on glioma cell membranes was recognized early, and subsequent studies have demonstrated their participation in tumor progression [15,21,22]. These extracellular nucleotides activate two distinct families of purinergic P2 receptors: ionotropic P2X receptors, which are calcium-permeable ion channels, and metabotropic P2Y receptors, which belong to the G-protein-coupled receptor superfamily [23,24]. To date, seven P2X subunits (P2X1-7) and eight P2Y receptors (P2Y_1,2,4,6,11–14_) have been cloned and characterized in humans based on their agonist sensitivity, sequence homology, and signal transduction mechanism. All P2X receptors are formed by the trimeric assembly of P2X subunits and mediate calcium influx upon ATP activation, although affinity, desensitization, and recovery properties vary among subtypes [25]. Regarding P2Y receptors, four subtypes are activated by adenine nucleotides (P2Y_1,11–13_), two exclusively respond to uracil derivatives (P2Y_6,14_), and two are activated by both adenine and uracil nucleotides (P2Y_2,4_) [26]. P2Y_12–14_ are coupled to Gi proteins, whereas P2Y_1,2,4,6,11_ are coupled to Gq/11-proteins that mediate calcium release from intracellular stores and diacyl-glycerol production.

Dysregulation of the purinergic system has been extensively documented in astrocytoma and glioblastoma models. Rat C6 glioma, a cell line that has been extensively utilized to study glioma biology, has been reported to release ATP [27] and express several P2Y receptor subtypes, such as P2Y_1_, P2Y_2_, and P2Y_12_, which are believed to influence their proliferation and differentiation [28,29]. In addition, P2X4 and P2X7 receptors are also expressed in this cell line and may contribute to mechanisms of cell death [30,31]. Moreover, ATP has been shown to induce chemokine production in C6 glioma cells, potentially enhancing cell motility and facilitating microglial recruitment to the tumor microenvironment, processes that may support tumor progression [32]. Notably, temozolomide, a DNA-alkylating agent used in the treatment of both primary and recurrent high-grade gliomas, has been reported to trigger an ATP surge associated with autophagy in U251 glioma cells, a response that appears to confer cytoprotection and may underlie mechanisms of therapeutic resistance [33]. Furthermore, carnosine has been shown to inhibit the proliferation of cells derived from human malignant gliomas, and more recent findings indicate that it suppresses ATP production in both patient-derived glioma samples and the T98G human glioma cell line [34].

Although many of the mechanisms described in gliomas broadly may be applicable to, or extrapolated as hypotheses for, oligodendrogliomas, there are currently no experimental studies that specifically and exclusively address oligodendrogliomas in relation to purinergic system. To address this critical knowledge gap, the present study aimed to systematically characterize the expression profile of functional P2X and P2Y purinergic receptors in the human oligodendroglioma (HOG) cell line.

## 2. Materials and Methods

### 2.1. Antibodies and Reagents

Primary antibodies against P2X and P2Y receptors were obtained from Alomone Labs (Jerusalem, Israel, cat. no. APR-002 for P2X4, APR-005 for P2X5, APR-004 for P2X7, APR-009 for P2Y_1_, APR-010 for P2Y_2_, APR-006 for P2Y_4_, and APR-012 for P2Y_12_). Poly-L-lysine and primary antibody against α-tubulin were from Sigma-Aldrich (St. Louis, MO, USA, cat. no. P8920 and T5168, respectively). Ki-67 primary antibody (clone SP6) was from Thermo Fisher Scientific (Waltham, MA, USA, cat. no. RM-9106-S). HRP-conjugated goat anti-rabbit secondary antibody was purchased from Dako (Barcelona, Spain, cat. no. P0448). Fluorescent secondary antibodies included Cy3™-conjugated donkey anti-rabbit IgG (Jackson ImmunoResearch, West Grove, PA, USA, cat. no. 711-165-452) and Alexa Fluor 488-conjugated goat anti-mouse IgG (Molecular Probes, Eugene, OR, USA, cat. no. A11001). ATP (cat. no. A7699), UTP (cat. no. U6625), diuridine tetraphosphate (Up_4_U, cat. no. D1262), and 2-aminoethoxydiphenyl borate (2-APB, cat. no. D9754) were purchased from Sigma-Aldrich. 2-methyl-thio-ADP (2MeSADP, cat. no. 1624) and MRS2179 (cat. no. 0900) were from Tocris Bioscience (Bristol, UK). The calcium indicator Fura-2 AM was obtained from Thermo Fisher Scientific (cat. no. F1221). Dulbecco’s Modified Eagle Medium (DMEM) was from Sigma-Aldrich (cat. no. D6546). GlutaMAX^®^, penicillin/streptomycin and fetal bovine serum (FBS) were purchased from Thermo Fisher Scientific (cat. no 35050-38, 15140-122, and 35050-083, respectively). The LIVE/DEAD^®^ viability/cytotoxicity assay kit and Hoechst 33342 were from Thermo Fisher Scientific (cat. no. L3224 and H1399, respectively). Primers, probes, and other analytical-grade chemicals were also acquired from Sigma-Aldrich.

### 2.2. Cell Culture

The human oligodendroglioma cell line (HOG) was purchased from Sigma-Aldrich (cat. no. SCC163). These cells originate from a single-cell clone established from a surgically excised human oligodendroglioma [35]. Functionally, HOG cells possess a variety of neurotransmitter receptors: A_2_-adenosine, prostaglandin E_1_, and β_2_-adrenergic receptors, all of which couple to the adenylate cyclase pathway, as well as muscarinic and histamine receptors that signal through inositol phosphate cascades [35,36]. HOG cells were maintained in DMEM, supplemented with GlutaMAX^®^, 10% heat-inactivated FBS, and penicillin/streptomycin. Cultures were incubated at 37 °C in a humidified atmosphere containing 5% CO_2_. To prevent culture overgrowth during the experimental period, time-lapse microscopy, cell proliferation and viability assays were performed in medium containing reduced serum (2% FBS).

### 2.3. Retrotranscription and Quantitative Real-Time PCR (qPCR)

Total RNA was isolated from cultured HOG cells using Speedtools total RNA extraction kit from Biotools (Madrid, Spain, cat. no. 21.212), following the manufacturer’s instructions as previously described [37]. Briefly, RNA samples were treated with TURBO DNase (Ambion, Austin, TX, USA, cat. no. AM1907), quantified, and reverse-transcribed using M-MLV reverse transcriptase, 6 μg random primers, and 350 μM dNTPs, all from Thermo Fisher Scientific (cat. no. 28025-013, 48190-011, and 10297-018, respectively). Quantitative real-time PCR reactions were carried out using LuminoCt^®^ qPCR readymix (Sigma-Aldrich, cat. no. L6669), 5 µL of the cDNA generated, gene-specific primers, and probes. Fast thermal cycling was performed using a StepOnePlus™ Real-Time PCR System (Applied Biosystems, Waltham, MA, USA) as follows: pre-denaturation at 95 °C for 20 s, followed by 40 cycles each of 95 °C for 1 s and 60 °C for 20 s. The results were normalized by parallel amplification of the endogenous control GAPDH. Relative mRNA expression levels were quantified using the comparative ΔΔCt method (2^−ΔΔCt^), as previously described by Livak and Schmittgen [38]. Detailed oligonucleotide and probe sequences, target genes, and product lengths are provided in Supplementary Table S1.

### 2.4. Western Blotting

HOG cells were lysed in ice-cold buffer containing 50 mM Tris-HCl, 150 mM NaCl, 1% Nonidet P-40, and Complete™ protease inhibitors (Roche Diagnostics, Barcelona, Spain, cat. no. 04693116001) at pH 7.4. After centrifugation, proteins were resolved by SDS-PAGE (10%) and transferred to PVDF membranes (GE Healthcare, Madrid, Spain, cat. no. A29433753). Membranes were blocked for 1 h at room temperature in TBS-T (10 mM Tris-HCl, 100 mM NaCl, 0.1% Tween-20; pH 7.5) containing 5% non-fat dry milk and incubated overnight at 4 °C with primary antibodies against P2X4 (1:500), P2X5 (1:200), P2Y_1_ (1:200), P2Y_2_ (1:200), P2Y_4_ (1:500), and P2Y_12_ (1:200). Blots were washed and incubated overnight at 4 °C with HRP-conjugated secondary antibodies (1:5000). Protein bands were detected using an enhanced chemiluminescence substrate (Thermo Fisher Scientific, cat. no. 34577) and visualized using an Image Quant LAS 500 imaging system (GE Healthcare).

### 2.5. Immunocytochemistry

HOG cells cultured on coverslips (250,000 cells/well) were fixed in 4% paraformaldehyde (PFA) for 15 min and permeabilized with 0.1% Triton X-100. After blocking for 1 h with 5% goat serum (Sigma-Aldrich, cat. no G9023) and 10% FBS, cells were incubated for 1 h at room temperature with primary antibodies against P2X4 (1:100), P2X5 (1:50), P2Y_1_ (1:50), P2Y_2_ (1:50), P2Y_4_ (1:100), and α-tubulin (1:1000), all diluted in blocking buffer. Secondary antibodies, Cy3™-conjugated donkey anti-rabbit IgG and Alexa Fluor 488-conjugated goat anti-mouse IgG, were applied 1:500 for 1 h at room temperature. Nuclei were counterstained with DAPI (Sigma-Aldrich, cat. no D9542). Coverslips were mounted with FluorSave™ (Merck, Darmstadt, Germany, cat. no. 345789), and confocal images were acquired using a Leica TCS SPE system (Leica Microsystems, Wetzlar, Germany).

### 2.6. Calcium Imaging in Single Cells

HOG cells were seeded on poly-L-lysine-coated coverslips (250,000 cells/well in 35 mm dishes) and loaded with 5 μM Fura 2-AM for 45 min at 37 °C in Locke’s solution (140 mM NaCl, 4.5 mM KCl, 2.5 mM CaCl_2_, 1.2 mM KH_2_PO_4_, 1.2 mM MgSO_4_, 5.5 mM glucose, 10 mM HEPES; pH 7.4). Coverslips were placed in a superfusion chamber (Warner Instruments, Hamden, CT, USA) mounted on a Nikon Eclipse TE-2000-E inverted microscope (Nikon, Madrid, Spain) and perfused continuously (1.5 mL/min) with Locke’s solution. Calcium imaging was carried out as previously reported [39,40]. Cells were stimulated for 40 s with various purinergic agonists: ATP (ranging from 10 nM to 1 mM, depending on the experiment), 2MeSADP (10 µM), Up_4_U (10 µM), and UTP (10 µM). In some experiments, 2-APB (100 µM) was applied 5 min prior to agonist stimulation.

Excitation wavelengths (340/380 nm) were selected using an Optoscan monochromator (Cairn), and fluorescence was recorded with an ORCA-ER CCD camera (Hamamatsu, Barcelona, Spain) controlled by Metafluor 6.3r6 software. Images were acquired every 250 ms with <5 ms switching time, and the F_340/F380_ ratio was calculated after background subtraction. Results are shown as normalized fluorescence ratios reflecting intracellular [Ca^2+^] changes.

### 2.7. Time-Lapse Videomicroscopy

Live-cell imaging was carried out using a Nikon Eclipse TE-2000 microscope under controlled environmental conditions (37 °C, 8% CO_2_), as previously described [41,42]. Phase-contrast images were captured every 5 min over a period of 4–5 days using a long-working-distance 20× phase-contrast objective (Nikon), a Zyla sCMOS camera (ANDOR), and NIS-Elements software (v4.7, Nikon). Cell tracking and lineage reconstruction were performed using tTt software (The Tracking Tool [43]), which enabled accurate monitoring of selected cells over time. Time-lapse sequences were compiled into movies using ImageJ software (version 1.42q, NIH, Bethesda, MD, USA).

### 2.8. Cell Proliferation Assay

HOG cells were seeded into 24-well plates at a density of 10,000 cells/well and incubated overnight to allow cell adherence. The following day, the culture medium was replenished, and cells were treated with the indicated pharmacological compounds for 72 h. Following treatment, cells were fixed in 4% PFA and processed for immunofluorescence staining, as previously described. Cellular proliferation was evaluated using a rabbit anti-Ki-67 primary antibody (1:100), a well-established marker of cellular proliferation. Cells were co-stained with anti-α-tubulin (1:1000) antibody, and nuclei were counterstained with DAPI. Coverslips were mounted, and confocal images were acquired using a Leica TCS SPE system.

### 2.9. Viability Assay

To evaluate cell viability, HOG cells were plated in 24-well culture plates at a density of 10,000 cells per well and allowed to attach overnight. The following day, the culture medium was replaced, and cells were exposed to the indicated pharmacological treatments for 72 h. Cell survival was subsequently determined using the LIVE/DEAD^®^ viability/cytotoxicity kit in accordance with the manufacturer’s instructions. Briefly, treated cells were washed with warm PBS and incubated with 2 µM Calcein-AM and 4 µM Ethidium homodimer-1 (EthD-1) in Locke’s solution for 30 min at 37 °C in the dark. Nuclei were also counterstained with 4 µM Hoechst 33342. Fluorescent signals corresponding to live cells (green), dead cells (red), and total nuclei (blue) were immediately captured using a Nikon Eclipse TE-2000 microscope, and the percentages of dead cells (%EthD-1–positive cells relative to total cells) was calculated from representative optical fields per condition.

### 2.10. Statistical Analysis

Data are presented as mean ± SEM from a minimum of three independent experiments. Statistical analysis was performed using GraphPad Prism 10 (GraphPad Software, San Diego, CA, USA). Differences between groups were analyzed using one-way ANOVA followed by Tukey’s post hoc test. A *p*-value < 0.05 was considered statistically significant.

## 3. Results

### 3.1. Characterization of Functional Purinergic Receptors in HOG Cells

The functional characterization of purinergic receptors was performed by analyzing changes in intracellular free calcium concentration ([Ca^2+^]_i_) elicited by the endogenous agonist ATP in HOG cells (Figure 1A). For these experiments, HOG cells were cultured on coverslips for 24 h to allow adhesion and were subsequently loaded with the membrane-permeable calcium indicator Fura-2 AM. The coverslips were then placed in a superfusion chamber mounted on an inverted microscope. Then, HOG cells were stimulated with increasing concentrations of ATP, and the changes in [Ca^2+^]_i_ were monitored at the single-cell level. Application of 1 µM ATP was sufficient to elicit a robust calcium response. The maximal response was achieved following stimulation with 10 µM ATP; therefore, this concentration was selected for all subsequent experiments (Figure 1B,C).

To determine the source of the intracellular calcium rise, HOG cells were treated with 10 µM 2-APB, an inhibitor of the inositol trisphosphate (IP_3_) receptor that mediates calcium release from the endoplasmic reticulum. Interestingly, 2-APB significantly attenuated, but did not completely abolish, the calcium response induced by 10 µM ATP (Figure 1D,E). This result indicates that the ATP-induced increase in [Ca^2+^]_i_ depends on both extracellular calcium influx and mobilization from intracellular stores. These findings suggest the involvement of both ionotropic P2X and metabotropic P2Y receptors in the ATP-mediated calcium response in HOG cells.

### 3.2. Identification of Purinergic P2X Receptors in HOG Cells

To characterize the expression profile of ionotropic P2X receptors in HOG cells, we analyzed both mRNA and protein levels of all known P2X subunits. Reverse transcription followed by qPCR revealed that P2X4 and P2X5 transcripts were the most abundantly expressed, whereas mRNAs for P2X1, P2X2, P2X3, P2X6, and P2X7 were nearly undetectable (Figure 2A).

Western blot analysis using subunit-specific antibodies confirmed the robust expression of P2X4 and P2X5 proteins in HOG cells (Figure 2B). According to UniProtKB predictions [44], the expected molecular weights for unglycosylated and glycosylated P2X4 proteins range from 40–45 kDa and 43–55 kDa, respectively. While the ranges for P2X5 are 44–47 kDa (unglycosylated) and 49–54 kDa (glycosylated). In our assays, P2X4 migrated at ~44 kDa and P2X5 at ~47–49 kDa, consistent with their unglycosylated canonical forms. Notably, P2X7 receptor (~69 kDa) was undetectable at both the mRNA and protein levels. Immunocytochemistry further confirmed the presence of P2X4 and P2X5 receptors, with immunostaining predominantly localizing to the cytoplasm (particularly the perinuclear region) and the plasma membrane (Figure 2C).

Due to the lack of selective agonists for P2X4 and P2X5, their functional contribution was interfered from the ATP-induced calcium responses recorded in the presence of 2-APB (Figure 1D,E).

### 3.3. Identification of Purinergic P2Y Receptors in HOG Cells

To identify the metabotropic purinergic receptors expressed in HOG cells, we first assessed the transcript levels of P2Y receptor subtypes via qPCR. HOG cells were found to predominantly express P2Y_1_ and P2Y_2_ receptor transcripts, both of which encode Gq/11-coupled receptors that mobilize intracellular calcium upon activation. Minor amounts of P2Y_4_ and P2Y_12_ transcripts were also detected, whereas P2Y_6_, P2Y_10_, P2Y_11_, P2Y_13_, and P2Y_14_ transcripts remained undetectable (Figure 3A).

Western blot analysis confirmed the expression of P2Y_1_ and P2Y_2_ proteins in HOG cells (Figure 3B). Based on UniProtKB predictions [44], the unglycosylated forms of these receptors are expected to migrate at ~41 kDa, whereas glycosylated variants range between 42–47 kDa. Consistent with these values, Western blot analysis revealed P2Y_1_ and P2Y_2_ bands at approximately 41 kDa, along with an additional prominent P2Y_1_ band at ~44 kDa, likely corresponding to glycosylated state. P2Y_4_ protein was also detected by Western blot despite its weak transcript signal, appearing at the expected band at ~41 kDa. Notably, although the P2Y_12_ receptor is widely recognized for promoting cell proliferation and microglial recruitment in high-grade gliomas [29], only low levels of P2Y_12_ mRNA were detected, while P2Y_12_ protein (~39 kDa) remained undetectable in the HOG oligodendroglioma cell line. Immunocytochemistry confirmed the presence of P2Y_1_, P2Y_2_, and P2Y_4_ receptors in HOG cells, revealing a subcellular distribution across both the cytosol and the plasma membrane (Figure 3C).

To evaluate receptor functionality, calcium imaging was performed in HOG cells loaded with Fura-2 AM. Cells were exposed to 10 µM 2MeSADP, a potent and selective P2Y_1_ receptor agonist [45]. As shown in Figure 4, the stimulation with 2MeSADP induced a robust increase in [Ca^2+^]_i_, although this transient was smaller than that triggered by 10 µM ATP, where both ionotropic and metabotropic components contribute to the response. Furthermore, cells stimulated with 10 µM Up_4_U or UTP, both agonists for P2Y_2_/P2Y_4_ receptors [46], also exhibited [Ca^2+^]_i_ elevations, which were lower in magnitude than those induced by 2MeSADP (Figure 4). These results confirmed the expression of functional P2Y_1_, P2Y_2_, and P2Y_4_ receptors in HOG cells, with the P2Y_1_ subtype acting as the main mediator of the metabotropic calcium response.

### 3.4. P2Y_1_ Receptor Modulates Proliferative Capacity, Migratory Behaviour and Survival of HOG Cells

Purinergic signalling through the P2Y_1_ receptor has been predominantly characterized as a driver of tumor progression, aggressive phenotypes, and microenvironment remodeling, particularly in astrocytoma and glioblastoma models [14,28,32,47]. To investigate the role of P2Y_1_ receptor activation in regulating HOG cell dynamics, we performed time-lapse videomicroscopy and single-cell tracking to monitor changes in proliferation, migration, and cell death over 72 h. HOG cells were cultured in complete DMEM supplemented with 2% FBS and either left untreated or exposed to the P2Y_1_-selective agonist 2MeSADP (10 µM) in the presence or absence of the selective antagonist MRS2179 (10 µM). Images were acquired every 5 min, and lineage trees illustrating the dynamics of HOG cells and their progeny were generated by single-cell tracking (Figure 5A).

Quantitative analysis of HOG cell lineage progression revealed a significant decrease in proliferative capacity, measured as the average number of cell divisions within each lineage tree analyzed, following treatment with the selective P2Y_1_ receptor agonist 2MeSADP. Moreover, this reduction was completely reversed when HOG cells were pre-incubated with the selective P2Y_1_ receptor antagonist MRS2179, which significantly increased proliferation above control levels (Figure 5B). These pharmacological effects were further validated by Ki-67 immunostaining, a well-established marker of cellular proliferation (Figure 6A,B). Furthermore, this antiproliferative mechanism appears to depend on P2Y_1_-mediated intracellular calcium mobilization, as pretreatment with the IP_3_ receptor antagonist 2-APB completely blocked the antiproliferative effect of 2MeSADP (Figure 6C). Consistent with these findings, analysis of cell cycle length showed that treatment with 2MeSADP increased the duration of the cell cycle, although this effect did not reach statistical significance. This tendency was significantly reversed by pre-treatment with MRS2179 (Figure 5C), further supporting a role for P2Y_1_ receptor signalling in the regulation of HOG cell proliferation.

Next, the effect of P2Y_1_ receptor activation on the migratory capacity of HOG cells was evaluated, as enhanced migratory behavior is widely associated with increased tumor invasiveness [47]. Treatment with 2MeSADP reduced the migration speed of HOG cells, whereas MRS2179 partially counteracted this reduction. However, neither effect reached statistical significance (Figure 5D). Finally, we investigated the effect of P2Y_1_ receptor activation on HOG cell viability by quantifying the percentage of cell death during live-cell imaging. As shown in Figure 5E, cell death was a rare event under control culture conditions. In contrast, activation of the P2Y_1_ receptor with 2MeSADP significantly increased the proportion of HOG cells undergoing cell death throughout the live imaging experiments. This effect was completely reversed by pre-treatment with MRS2179, further supporting the specificity of the P2Y_1_ receptor-mediated response. To validate this observation, we performed a LIVE/DEAD assay using calcein-AM and ethidium homodimer-1 (EthD-1) to quantify the percentage of HOG dead cells following 2MeSADP treatment. As depicted in Figure 6E,F, treatment with the P2Y_1_ agonist significantly increased the percentage of EthD-1-positive cells, confirming that P2Y_1_ receptor activation compromises the viability of cultured HOG cells. Consistent with our live-imaging results, pre-treatment with MRS2179 successfully rescued the cytotoxicity induced by 2MeSADP. However, when attempting to block intracellular calcium release, 2-APB pre-treatment alone induced a remarkable basal increase in cell death compared to control cells. Due to this intrinsic toxicity, subsequent application of 2MeSADP did not further exacerbate cytotoxicity, preventing a clear assessment of calcium signaling involvement in this specific viability assay.

Taken together, these findings indicate that activation of the P2Y_1_ receptor exerts multiple antitumor effects on HOG cells in vitro, including reduced proliferative capacity, a tendency towards decreased migratory behavior, and diminished cell viability, supporting the potential of P2Y_1_ receptor signalling as a therapeutic target in human oligodendroglioma cells.

## 4. Discussion

The purinergic signalling system has emerged as a key regulator of brain tumor pathophysiology, modulating neural cell proliferation, differentiation, cell death, and tumor microenvironment remodeling [12,14,15]. While extensive literature addresses ATP-mediated signalling in glioblastoma and astrocytic models, its role in oligodendrogliomas has remained unexplored until now. To our knowledge, this study provides the first systematic characterization of functional purinergic receptors in a human oligodendroglioma in vitro model, demonstrating that selective activation of the P2Y_1_ receptor exerts potent antitumor effects by inhibiting cell proliferation, slowing migration, and inducing cell death.

Our findings demonstrate that the HOG human oligodendroglioma cell line expresses a distinct subset of purinergic receptors, including ionotropic P2X4 and P2X5, and metabotropic P2Y_1_, P2Y_2_, and P2Y_4_ receptors. Calcium imaging experiments revealed that extracellular ATP elicits transient increases in intracellular calcium concentration through a dual mechanism: extracellular Ca^2+^ influx via ionotropic P2X channels and Ca^2+^ mobilization from intracellular stores via G-protein-coupled P2Y receptors, as confirmed by partial sensitivity to the IP_3_ receptor antagonist 2-APB. Unlike high-grade astrocytomas and glioblastomas, where P2X7 expression is frequently elevated and linked to invasive phenotypes, neuroinflammation, and resistance to apoptosis [30,31], HOG cells showed no detectable P2X7 expression at either the mRNA or protein level. Notably, P2X7 shares the highest degree of similarity with P2X4 receptor, representing the two most closely related P2X subunits in terms of amino acid sequence, chromosomal localization, and overlapping tissue distribution [48]. Evidence from an alveolar cell line suggests that P2X4 and P2X7 receptors undergo reciprocal cross-regulation, wherein P2X7 knockdown upregulates P2X4 expression, while P2X4 depletion conversely increases P2X7 levels [49]. Given that P2X4 possesses a markedly higher affinity for ATP compared to P2X7 [25], the prominent expression of P2X4 receptors in HOG cells may reflect an advantageous functional adaptation in oligodendrogliomas.

On the other hand, the P2Y_12_ receptor was not expressed in HOG cells, despite being frequently implicated in glioma cell proliferation and microglial recruitment in high-grade gliomas [29]. It is therefore likely that the high abundance of P2Y_1_ and P2Y_2_ receptors establishes these metabotropic subtypes as the primary mediators of adenine and uracil nucleotide responses in oligodendroglioma cells. The role of the P2Y_1_ receptor in gliomas has historically yielded complex and context-dependent results. In astrocytomas and glioblastomas, P2Y_1_-dependent signalling has been predominantly associated with pro-tumoral effects, including stimulation of cell proliferation [28], induction of chemokine secretion leading to microglial chemoattraction [32], and promotion of cell motility [47]. In contrast, our studies demonstrate that P2Y_1_ receptor activation in human oligodendroglioma cells mediates a strikingly beneficial, tumor-suppressive phenotype. Stimulation with the selective P2Y_1_ agonist 2MeSADP significantly reduced the mean number of cell divisions across tracked cell lineages and induced a trend toward cell cycle elongation. Moreover, P2Y_1_ activation increased cell death and tended to decrease mean migration velocity, pointing to an attenuation of the invasive potential typically associated with diffuse glioma progression [47]. Crucially, all observed inhibitory actions of 2MeSADP were fully rescued by pre-treating cells with the selective P2Y_1_ antagonist MRS2179.

Despite these promising findings, the present study has certain limitations that warrant consideration. Primarily, our experimental approach relied on the HOG cell line in vitro model. While this provided a robust and well-defined system for the initial functional characterization of these receptors, it inherently lacks the complex cellular heterogeneity and intact tumor microenvironment found in vivo. Therefore, future studies must aim to validate this unique purinergic expression profile, particularly the absence of P2X7 and the therapeutic vulnerability of P2Y_1_, in different oligodendroglioma cell lines, primary patient-derived oligodendroglioma cultures, and in vivo orthotopic xenograft models. Additionally, while we have established the functional phenotypic consequences of P2Y_1_ activation, the precise downstream intracellular signaling cascades mediating the observed reduction in cell proliferation, attenuated migration, and induction of cell death remain to be fully elucidated. Future investigations detailing these specific molecular pathways, alongside studies exploring the potential synergistic effects of P2Y_1_-targeted therapies in combination with standard chemoradiation regimens, will be crucial steps toward translating these foundational insights into viable clinical strategies for oligodendroglioma patients.

## 5. Conclusions

In conclusion, our findings establish that the HOG human oligodendroglioma cell line harbors a functional purinergic receptor machinery and highlight the P2Y_1_ receptor as an attractive pharmacotherapeutic target in oligodendroglioma. Unlike classical high-grade gliomas, where P2Y_1_ inhibition might be desired to limit migration, HOG cells appear to be vulnerable to P2Y_1_ activation, which acts directly to limit expansion and trigger cell death.

## Figures and Tables

**Figure 1:**
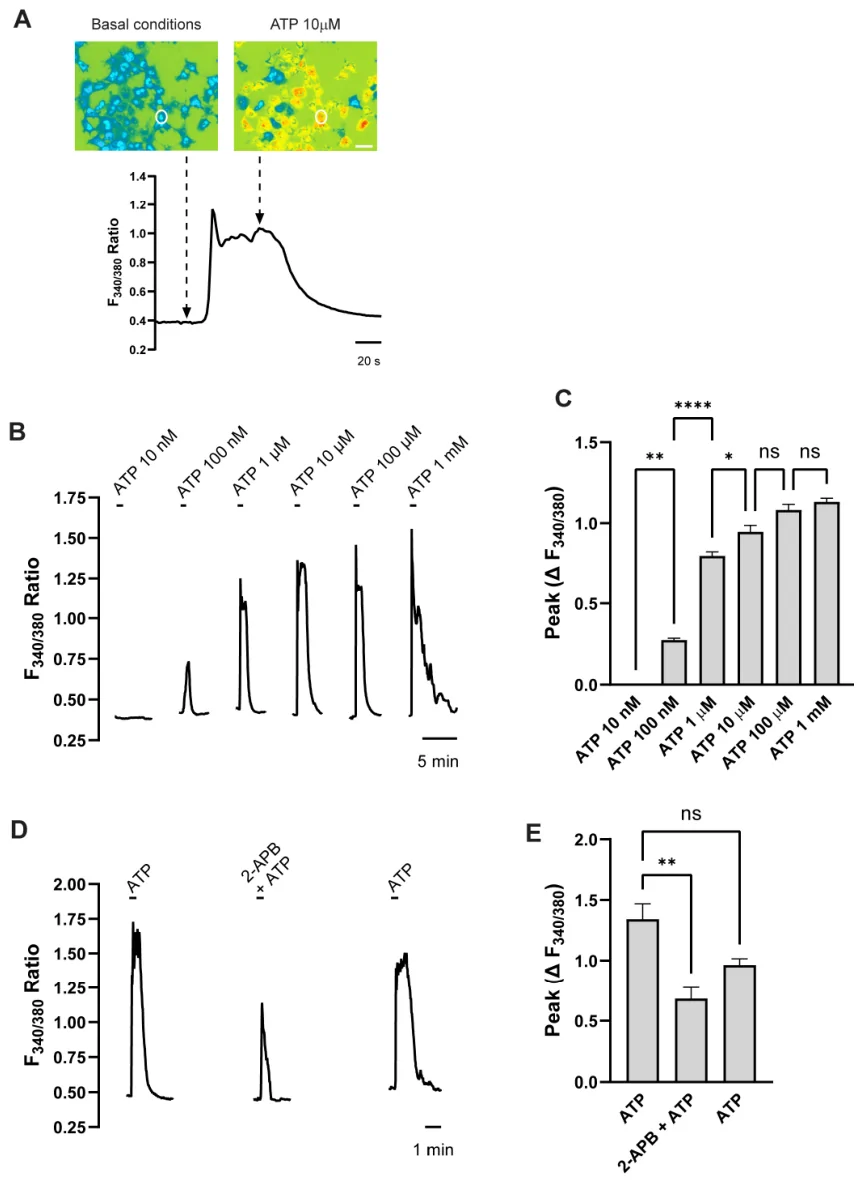
**Functional characterization of purinergic responses and calcium signaling sources in HOG cells.** (**A**) Representative pseudocolor fluorescence microphotographs of Fura-2 AM-loaded HOG cells under basal conditions and during 10 µM ATP stimulation (top), alongside corresponding single-cell (white circle) trace monitoring changes in the F_340/380_ fluorescence ratio over time (bottom). Scale bar = 50 µm. (**B**) Representative traces illustrating changes in the F_340/380_ ratio elicited by 30 s application of increasing concentrations of ATP ranging from 10 nM to 1 mM. The time labels indicate the time scale of the X-axis. (**C**) Concentration–response curve quantifying the maximal peak fluorescence changes ΔF_340/380_ induced by the different ATP concentrations. (**D**) Representative traces demonstrating the inhibitory effect of the IP_3_ receptor antagonist 2-APB (100 µM) on the 10 µM ATP-induced [Ca^2+^]_i_ transient. ATP applications lasted 30 s; the time labels indicate the time scale of the X-axis. (**E**) Quantification of the peak amplitude changes ΔF_340/380_ for ATP alone, in the presence of 2-APB + ATP, and following washout. Data are presented as mean ± SEM of 3 independent experiments. Statistical significance was determined using one-way ANOVA followed by Tukey’s multiple-comparison test. * *p* < 0.05, ** *p* < 0.01, **** *p* < 0.0001; ns, not significant.

**Figure 2:**
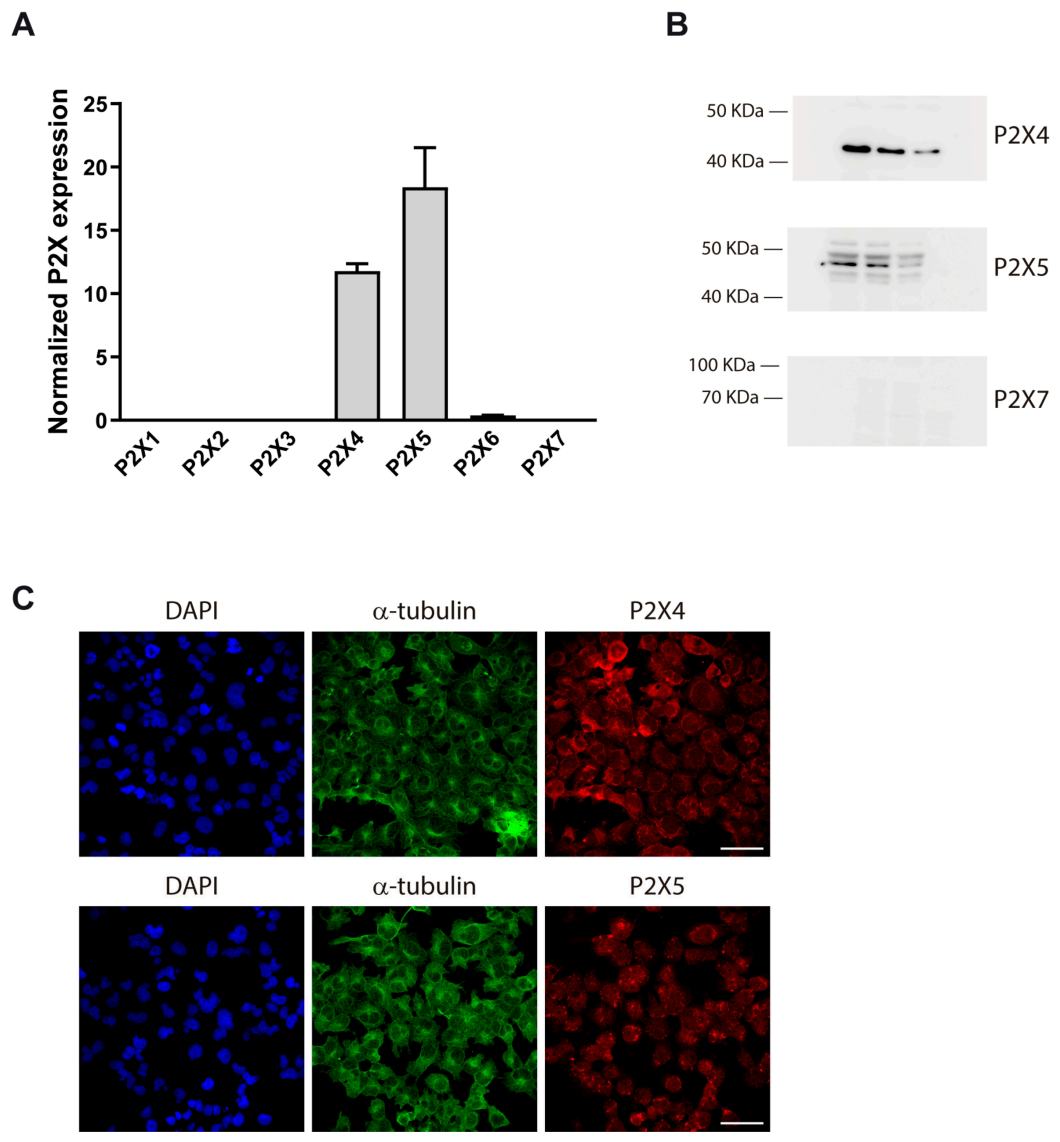
**Expression profile and subcellular localization of ionotropic P2X receptor subunits in HOG cells.** (**A**) Analysis of mRNA expression levels for P2X1 to P2X7 receptor subunits, demonstrating that P2X4 and P2X5 are the predominantly expressed transcripts in HOG cells, while the remaining subunits are nearly undetectable. Data are presented as mean ± SEM of 3 independent experiments. (**B**) Representative Western blot analysis confirming the protein expression of P2X4 and P2X5 receptors. Each lane represents an extract from independently cultured HOG cells collected at a distinct cell passage. Molecular weight markers are indicated on the left. (**C**) Representative immunocytochemical fluorescence micrographs showing the subcellular distribution of P2X4 (top panels) and P2X5 (bottom panels) receptors (red). Cells are counterstained with α-tubulin to visualize the cytoskeleton (green) and DAPI to label nuclei (blue). Scale bar = 50 µm.

**Figure 3:**
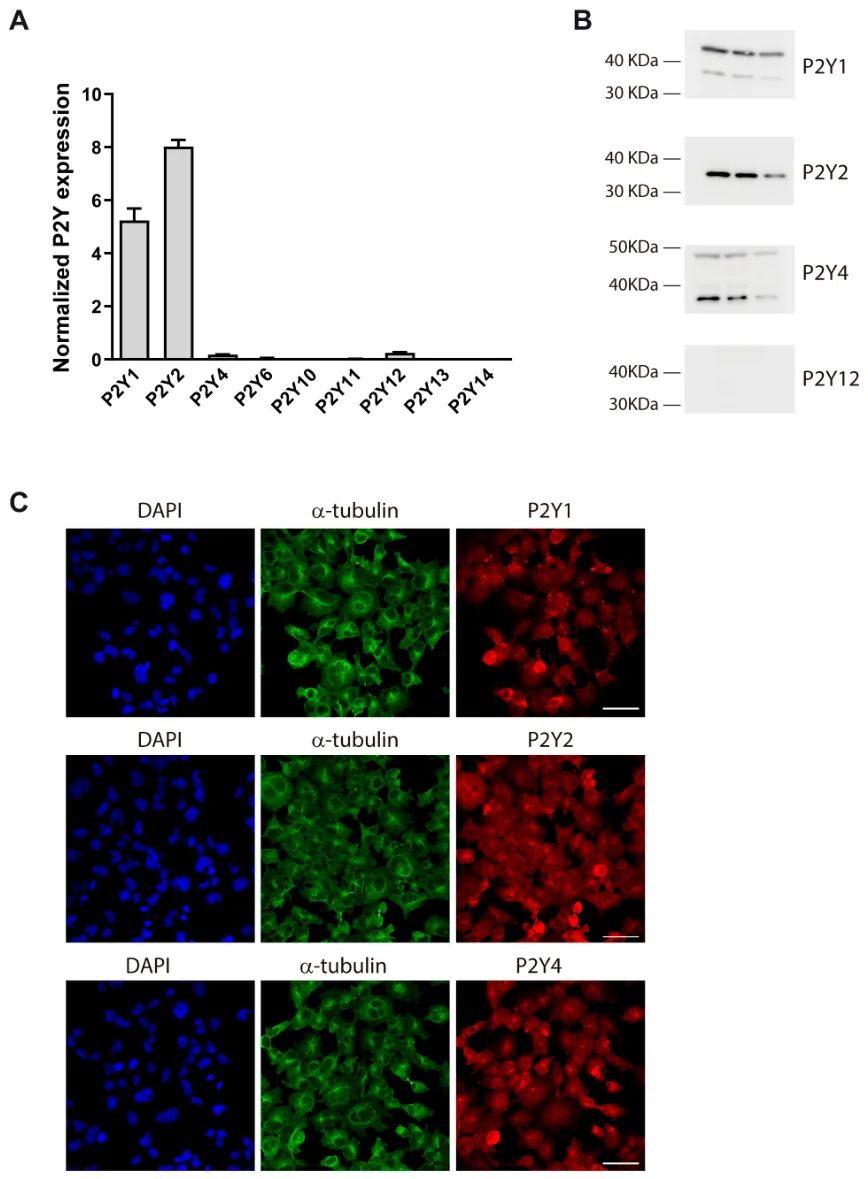
**Expression profile and subcellular distribution of metabotropic P2Y receptor subtypes in HOG cells.** (**A**) Screening of mRNA expression levels for P2Y_1_ to P2Y_14_ receptors, revealing predominant transcript expression of P2Y_1_ and P2Y_2_ subtypes. Data in (**A**) are presented as mean ± SEM of 3 independent experiments. (**B**) Representative Western blot analysis validating the protein expression of P2Y_1_, P2Y_2_, and P2Y_4_ receptors. Each lane represents an extract from independently cultured HOG cells collected at a distinct cell passage. Molecular weight markers are indicated on the left. (**C**) Representative immunocytochemical fluorescence micrographs showing the subcellular distribution of P2Y_1_ (top panels), P2Y_2_ (middle panels), and P2Y_4_ (bottom panels) receptors (red). Cells are counterstained with α-tubulin to visualize the cytoskeleton (green) and DAPI to label nuclei (blue). Scale bar = 75 µm.

**Figure 4:**
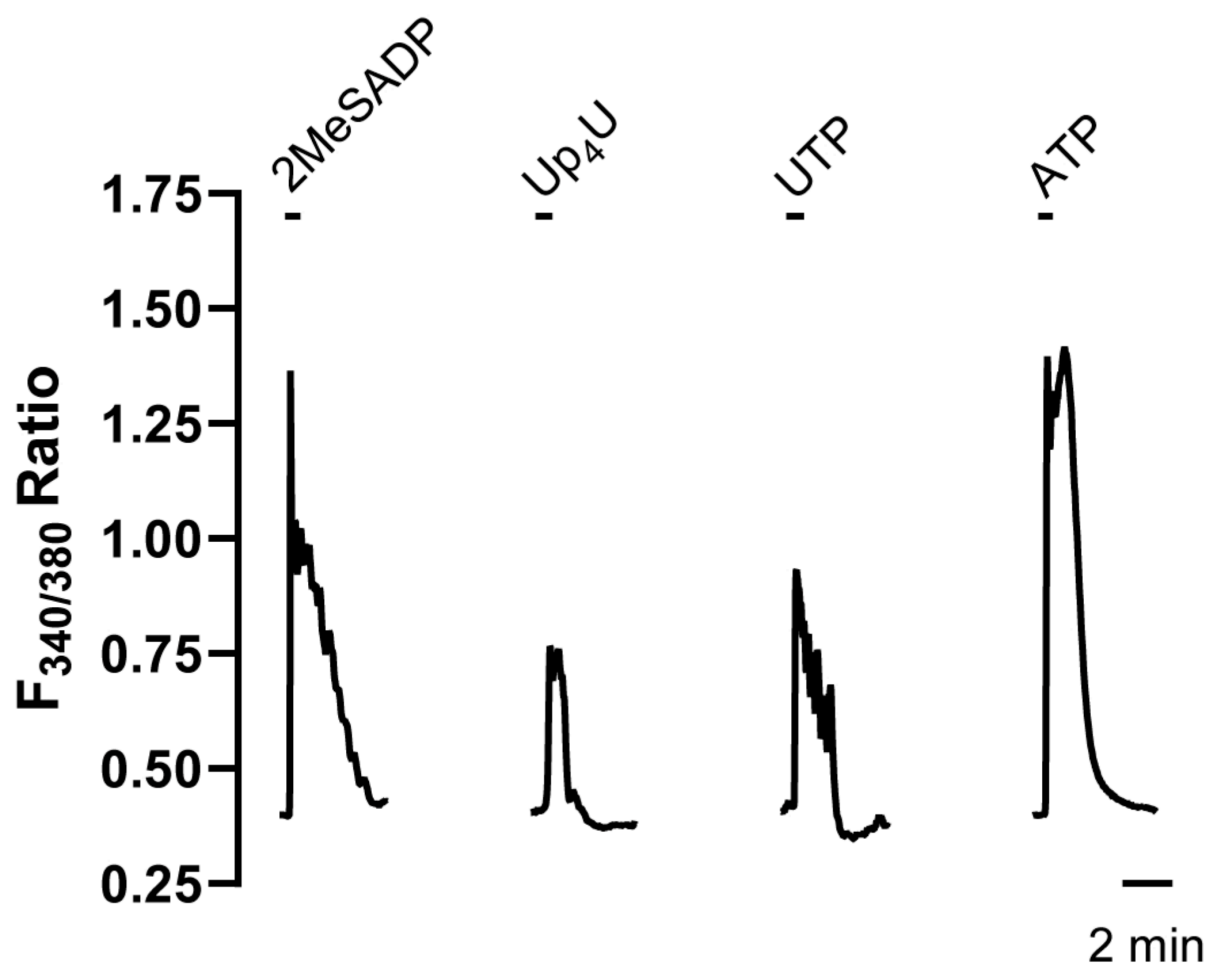
**Functional assessment of metabotropic P2Y receptor subtypes in HOG cells.** Representative single-cell calcium imaging traces illustrating changes in the F_340/380_ fluorescence ratio over time in Fura-2 AM-loaded HOG cells. The trace shows [Ca^2+^]_i_ transients elicited by the application of 10 µM 2MeSADP (a selective P2Y_1_ agonist), 10 µM Up_4_U, and 10 µM UTP (both dual P2Y_2_/P2Y_4_ agonists), compared against the total purinergic response triggered by 10 µM ATP. All agonist applications lasted 30 s; the time labels indicate the time scale of the X-axis.

**Figure 5:**
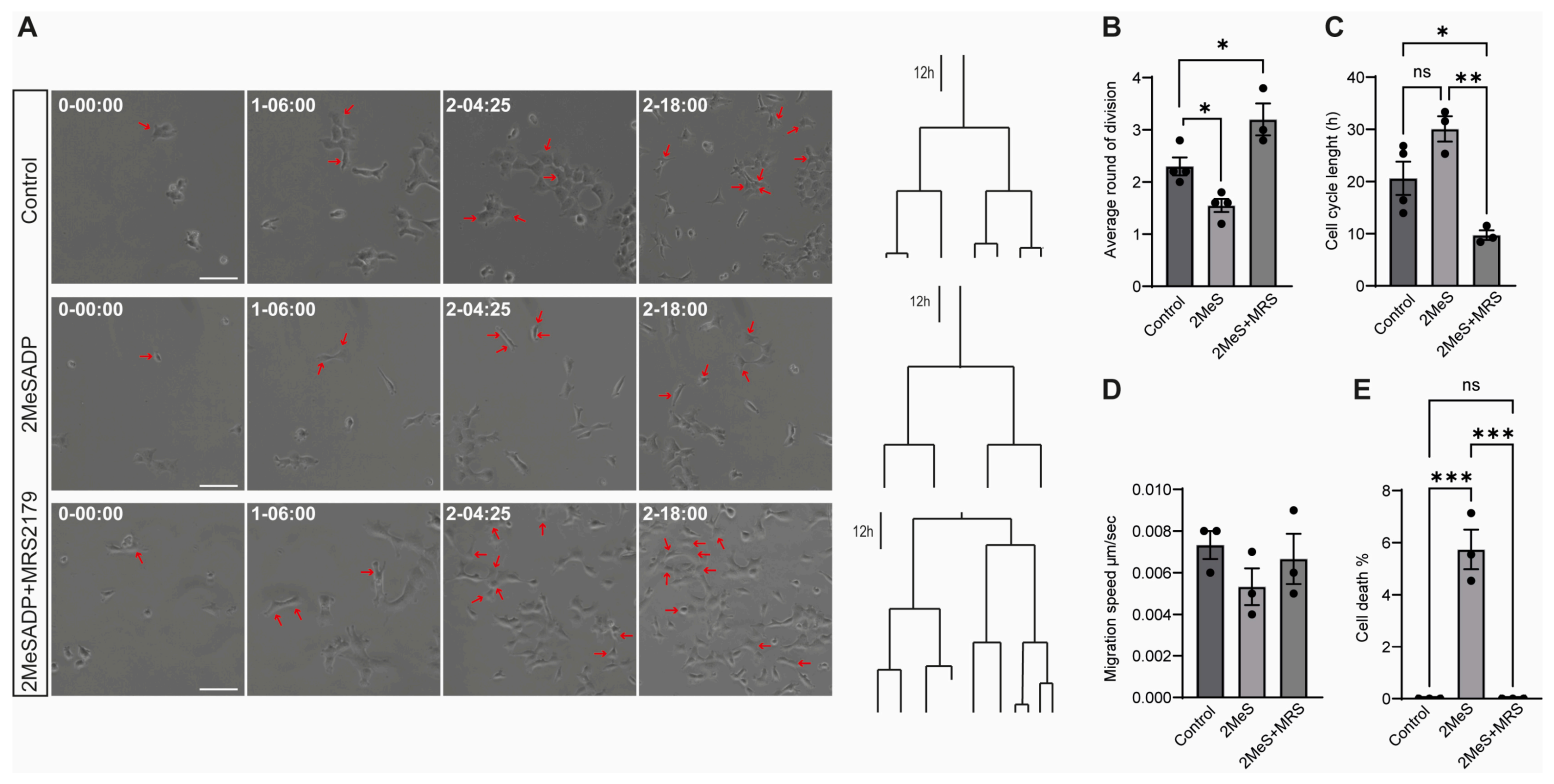
**P2Y_1_ receptor activation modulates proliferation dynamics, cell cycle length and cell viability in HOG cells.** (**A**) Representative time-lapse microscopy images (left) and lineage trees (right) showing the proliferative behaviour of HOG cells under control conditions, following treatment with the P2Y_1_ receptor agonist 2MeSADP (2MeS), or after co-treatment with 2MeSADP and the selective P2Y_1_ receptor antagonist MRS2179. Red arrowheads indicate representative cell division events. The phase contrast images depict the lineage progression in the live imaging experiment (day–hour–min). Scale bar = 100 µm. (**B**) Histogram depicting the quantification of the average number of cell divisions per cell. Note how activation of P2Y_1_ receptor significantly reduced the average number of divisions, whereas pharmacological blockade with MRS2179 restored proliferative activity. (**C**) Cell cycle length analysis showing that P2Y_1_ receptor activation significantly prolonged the cell cycle compared with control cells, while MRS2179 reversed this effect. (**D**) Histogram depicting the quantification of migration speed, which revealed no significant differences among treatments. (**E**) Histogram depicting the percentage of cell death during live-cell imaging. P2Y_1_ receptor activation significantly increased cell death, whereas co-treatment with MRS2179 prevented this effect, restoring cell viability to control levels. Data are presented as mean ± SEM. Individual dots represent independent experiments. Statistical significance was determined using one-way ANOVA followed by Tukey’s multiple-comparison test. * *p* < 0.05, ** *p* < 0.01, *** *p* < 0.001; ns, not significant.

**Figure 6:**
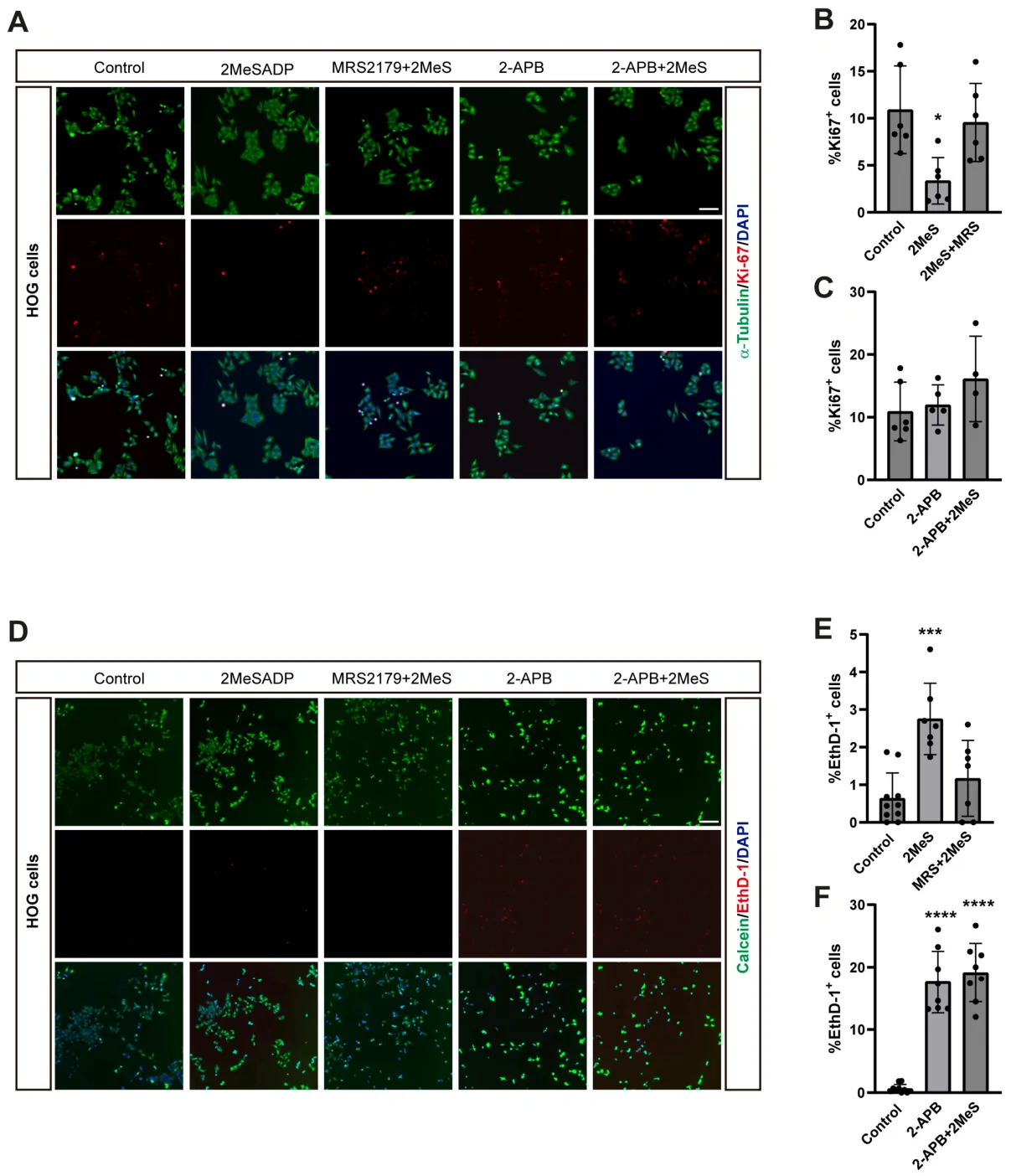
**Effect of P2Y_1_ receptor activation on proliferation and survival of HOG cells.** (**A**) Representative fluorescence micrographs showing HOG cell proliferation under control conditions or following treatment with the P2Y_1_ receptor agonist 2MeSADP (2MeS, 10 µM), either alone or in combination with the P2Y_1_ receptor antagonist MRS2179 (10 µM) or the IP_3_ receptor antagonist 2-APB (100 µM). Actively proliferating cells were labeled with Ki-76 (red), the cytoskeleton with α-tubulin t (green), and nuclei with DAPI (blue). Scale bar = 100 µm. (**B**) Quantification of the percentage of Ki-76-positive cells relative to total DAPI-positive cells. P2Y_1_ receptor activation significantly decreased proliferation, an effect prevented by MRS2179. (**C**) Quantification of cell proliferation in the presence of 2-APB. Blockade of endoplasmic reticulum prevented the 2MeSADP-induced decrease in proliferation. (**D**) Representative images of the LIVE/DEAD viability assay for the treatment groups described in (**A**). Live cells are stained with calcein-AM (green), dead cells with EthD-1 (red), and nuclei with Hoechst 33342 (blue). (**E**) Quantification of the percentage of EthD-1-positive dead cells relative to total Hoechst-positive cells. P2Y_1_ activation significantly increased cell death, which was reversed by MRS2179. (**F**) Quantification of cell death in the presence of 2-APB, demonstrating that 2-APB treatment alone is cytotoxic for HOG cells. Data are expressed as mean ± SEM. Individual dots represent independent experiments. Statistical significance was determined using one-way ANOVA followed by Tukey’s multiple-comparison test. * *p* < 0.05, *** *p* < 0.001, **** *p* < 0.0001.

## Data Availability

The raw data supporting the conclusions of this article will be made available by the authors on request.

## Supplementary Materials

The following supporting information can be downloaded at: https://www.mdpi.com/article/10.3390/biology15211969/s1, Table S1: Oligonucleotide primers and probe sequences used for real-time qPCR analysis.

## Abbreviations

The following abbreviations are used in this manuscript:

CNS	Central Nervous system
WHO	World Health Organization Classification
2MeSADP	2-Methyl-thio-ADP
Up_4_U	Diuridine tetraphosphate
2-APB	2-Aminoethoxydiphenyl borate
FBS	Fetal Bovine Serum
[Ca^2+^]_i_	Intracellular free calcium concentration
IP_3_	Inositol trisphosphate
