# Supplementary material for: Functional Characterization of Purinergic Signaling in HOG Human Oligodendroglioma Cell Line: Tumor-Suppressive Role of the P2Y1 Receptor
Source: Biology (Basel). 2026 Nov 2;15(21):1969. doi: 10.3390/biology15211969 (PMC13762438; doi:10.3390/biology15211969)
Supplement: Supplementary file 1 [file biology-15-01969-s001.zip › Supplementary Table S1.pdf]

Supplementary Table S1: Oligonucleotide primers and probes sequences used for real-time qPCR analysis.

Gene	Forward primer	Reverse primer	TaqMan Probe	Amplicon
P2rx1	TACGTGGTGCAAGAGTCAGG	CCAGGTCACAGTGCCAGTC	CATCACCA	91
P2rx2	GGAAGATGGGGCCTCTGT	GATGTTGCCCTTGGAGAAGT	CAGCATCC	112
P2rx3	GCGGCCTTTACTTCTGTGG	AAACTTCTTGGCTTTGTACTGGTC	TCCTGCTC	96
P2rx4	GCACAACGTATCTCCTGGCTA	TGCCATAGGCCTTGATGAG	CTGGCTGG	95
P2rx5	GTCAGAAGGGGAACGGATCT	GCAATTCACGTGCTCCTGT	GCTCCAGG	64
P2rx6	TGTGGATAGAGAAGCCCATTTC	AGAGTTGGCGGTTGCTTTC	CTGGAGGA	76
P2rx7	GCGGTTGTGTCCCGAGTAT	ACCTTCCGGTCTGAATTCCT	TTCCTCTG	107
P2ry1	ATGTTCTGTGTCCCCTTGGT	AAATCAAAGCTCTCACAATTAATCC	CTGGGCTG	64
P2ry2	TTGCACTGGGGGTAAATATGA	AGAAGGCAGACGATGGACA	CTCCTCCT	60
P2ry4	CTCTGGAGAGAAGCCCTCAC	GAGAGATGAGGTCACCCATGA	GGAGGCAG	68
P2ry6	CCTAGCCCTGGTCCATACG	ACAACCCTGATGTTGCTGTG	CTTCCAGC	68
P2ry10	TGCAGTATAAATAGCATAGACATTTGG	TTCATTCTTCCATAGTCACTCTGTTC	CTCCTCCC	99
P2ry11	GGGAACTGGGTAGCAGACAC	AGTTGGCAGGGCAGGACT	GCTGAGGA	86
P2ry12	TCCATTCAAAATTCTTAGTGATGC	CGGAGGTAACTTGACACACAAA	ACTGGGAA	74
P2ry13	CTGCCGCCATAAGAAGACA	GCATCACTGTGGTGTTCATTG	ACACTGGA	78
P2ry14	CCACTTCAAGACGACAAACG	GGAGGTTGAATTGATCATCTTGT	TTGCCCAG	125
